# A Systematic Review of the Role of Chimeric Antigen Receptor T (CAR-T) Cell Therapy in the Treatment of Solid Tumors

**DOI:** 10.7759/cureus.14494

**Published:** 2021-04-15

**Authors:** Raheel S Siddiqui, Muhammad Sardar

**Affiliations:** 1 Internal Medicine, Icahn School of Medicine at Mount Sinai (New York City Health and Hospitals/Queens), Jamaica, USA; 2 Internal Medicine, Monmouth Medical Center, Long Branch, USA

**Keywords:** car-t, chimeric antigen receptor (car) t-cell, solid tumor

## Abstract

Chimeric antigen receptor T (CAR-T) cell therapy utilizes patients' own T lymphocytes that are engineered to attack cancer cells. It is Food and Drug Administration (FDA)-approved in various hematological malignancies and currently being evaluated in solid cancers in early phase studies. We did a systematic review consisting of 15 prospective clinical trials (n=159) evaluating CAR-T cells in solid cancers. Early phase trials showed promising response rates in ovarian epithelial cancer (100%), human epidermal growth factor receptor 2* *(HER2)-positive sarcoma (67%), epidermal growth factor receptor (EGFR)-positive biliary tract cancer (65%), advanced gastric/pancreatic cancer (82%), hepatocellular carcinoma (67%), and colorectal cancer (70%). The median overall response across all malignancies was 62% (range 17%-100%). Median progression-free survival and overall survival were not reached in most trials. Cytokine release syndrome was seen in only one patient with cholangiocarcinoma who received EGFR-specific CAR-T cell therapy. Although survival data is still not mature, CAR-T cell therapy in solid malignancies did show encouraging response rates and was well-tolerated.

## Introduction and background

Traditionally, the standard modes of treatment for solid malignancies have included surgical resection, radiation therapy, and chemotherapy depending upon the type and stage of the disease. In patients with advanced and metastasized solid tumors, surgical options are very limited and often restricted to palliation rather than cure. The radiation therapy, given externally or internally to the desired location, can induce damage to the surrounding normal tissue due to exposure to ionizing radiation. The side effects of the chemotherapy depend on the type and location of the cancer and the dose and choice of chemotherapy [1]. Toward the second half of the 20th century and the start of the 21st century, extensive research has been done to explore different treatment strategies for the treatment of cancers. Newer approaches in the treatment of malignancies include monoclonal antibodies, checkpoint inhibitors, adoptive T cell therapies, and targeted therapies [2-4]. One of the most promising advances in the treatment of cancers is chimeric antigen receptor (CAR) T cell therapy, which is a hot topic for research since the first effective CAR-T cell was introduced in 2002 [5]. Chimeric antigen receptor T cell therapy is a form of cancer immunotherapy in which a patient’s T cells are modified outside the body to express specific tumor-specific antigens, which are then infused back into the body to attack the target cells. CAR-T cells attack the tumor-associated antigen in a major histocompatibility complex (MHC)-nonrestricted manner [6]. CARs consist of an extracellular antigen-binding region composed of a single-chain variable fragment from tumor-associated antigen (TAA) specific antibody, a spacer domain, a transmembrane domain, and an intracellular signaling tail that includes CD3z and one or more immunostimulatory domains necessary for T-cell activation and proliferation [7-8]. CAR-T cell therapy has shown clinical efficacy and safety in patients with hematological malignancies and is currently approved by the Food and Drug Administration (FDA) for the treatment of refractory/relapsed cases of acute lymphoblastic leukemia, diffuse large B-cell lymphoma, mantle cell lymphoma, follicular lymphoma, and multiple myeloma [9-14]. After initial success in the realm of hematological malignancies, CAR-T cell therapy is now extensively investigated for its role in the treatment of refractory and relapsed cases of solid malignancies after exhausting systemic treatment options. We carried out a systematic review of prospective clinical trials to evaluate the efficacy of CAR-T cell therapy in patients with advanced solid tumors who either failed to respond or relapsed despite receiving standard chemotherapy treatment.

## Review

Methodology

**Figure 1:**
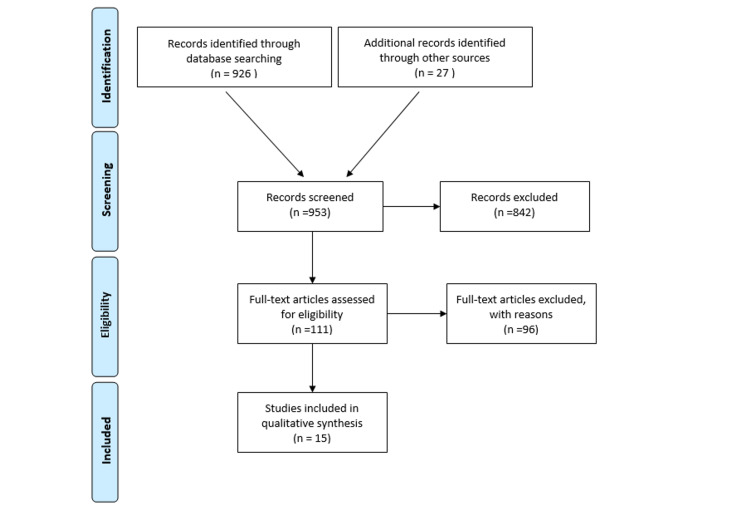
PRISMA flow diagram PRISMA: Preferred Reporting Items for Systematic Reviews and Meta-Analyses

The literature search was performed using the PubMed database and proceedings from the American Society of Clinical Oncology (ASCO). "Receptors, chimeric antigen" was the controlled vocabulary term (MeSH) used to search PubMed. CAR-T cell therapy was the keyword used to identify abstracts from ASCO proceedings. All data was included up to September 8, 2020, which was the day we concluded our search. We included all prospective clinical trials with outcome data available involving patients with solid malignancies treated with CAR-T cell therapy. Clinical trials in patients with hematological malignancies, case reports, case series, retrospective analysis, systematic reviews, and meta-analyses were excluded. We followed methods specified in the Preferred Reporting Items for Systematic Reviews and Meta-Analyses (PRISMA) statement for reporting systematic reviews (Figure 1) [15]. A total of 926 records from PubMed and 27 articles from ASCO meetings were identified after the initial search. After excluding duplicates and articles not meeting our inclusion criteria, 15 studies were selected for final analysis. The following variables were analyzed: type of study, number of patients, type of malignancy, stage, conditioning regimen used, and CAR-T product. The primary outcome of our study was overall response rate while secondary outcomes included complete response rate, partial response rate, disease stability, median progression-free survival, overall survival, grade 3 or higher neurotoxicity, and cytokine release syndrome.

Results

A total of 159 patients across 15 prospective clinical trials evaluating CAR-T cell therapy in various solid malignancies were included in our review. Table 1 shows a summary of included clinical trials. Overall response ranged from 17% to 100% across various malignancies. The median overall response rate was 62% (SD 0.23). The rate of disease stability ranged from 9%-100% across all trials with a median of 44% (SD 0.23). Median progression-free survival and overall survival were not reached in most trials. Cytokine release syndrome was seen in only one patient with cholangiocarcinoma who received epidermal growth factor receptor (EGFR)-specific CAR-T cell therapy.

**Table 1:** Summarizes the efficacy and safety of CAR-T cells in solid malignancies PSCA: prostate stem cell antigen; R/R: relapsed/refractory; CAR-T: chimeric antigen receptor T; EGFR: epidermal growth factor receptor; HER-2: human epidermal growth factor receptor 2; CEA: carcinoembryonic antigen; HAI: hepatic artery infusion

Author and Year	Phase of Trial	Type of Malignancy	Stage	CAR-T Cell Product	Condition Regimen	No. of Patients	Overall Response Rate	Complete Response	Partial Response	Stable Disease	Median Progression-Free survival	Grade 3 or 4 Neurotoxicity/CRS
Guo et al., 2017 [16]	1	Biliary tract cancers	4	EGFR-specific CAR-T cell	Nab-Paclitaxel and cyclophosphamide	17	11/17 (65 %)	1/17 (6%)	0	10/17 (59%)	4 m (range, 2.5-22 m)	1 CRS
Wallen et al., 2009 [17]	1	Metastatic melanoma	4	Mart-1, gp100, or tyrosinase-specific CAR-T	fludarabine	9	3/9 (33%)	0	1/9 (11%) with minor response (20-30 % reduction)	2/9 (22%)	2.1m	0
Adusumilli et al., 2019 [18]	1	Malignant pleural mesothelioma	2-4	Mesothelin-specific CAR-T cells	cyclophosphamide	14	11/14 (79%)	2/14 (14%)	5/14 (36%)	4/14 (29%)	NA	0
Zhan et al., 2019 [19]	1	Gastric and pancreatic adenocarcinoma	4	CLDN 18.2-specific CAR-T cell	Fludarabine and cyclophosphamide, with or without nab-paclitaxel	11	9/11 (82%)	1/11 (9%)	3/11 (27%)	5/11 (45%)	136 days (44-237)	0
Becerra et al., 2019 [20]	1	Pancreatic, gastric, or prostate cancers	4	PSCA-specific at CAR-T cell	Cyclophosphamide with or without fludarabine	13	8/13 (62%)	0	0	8/13 (62%)	NA	0
Hegde et al., 2017 [21]	1	Sarcomas	4	HER2-specific CAR-T cell	Fludarabine with or without cyclophosphamide	6	4/6 (67%)	2/6 (33%)	0	2/6 (33%)	NA	0
Tanyi et al., 2016 [22]	1	Epithelial ovarian cancer	3-4	Mesothelin-specific CAR-T cell	cyclophosphamide	6	6/6 (100 %)	0	0	6/6 (100 %)	NA	NA
Zhai et al., 2017 [23]	1	Hepatocellular carcinoma	R/R	GP3-specific CAR-T cell	Fludarabine + cyclophosphamide	6	4/6 (67%)	0	1/6 (17%)	3/6 (50%)	NA	0
Beatty et al., 2018 [24]	1	Pancreatic ductal adenocarcinoma	R/R	Mesothelin-specific CAR-T cell	No lymphodepletion	6	2/6 (33%)	0	0	2/6 (33%)	NA	0
Feng et al., 2017 [25]	1	Biliary tract and pancreatic carcinoma	R/R	HER2-specific CAR-T cell	nab-paclitaxel and cyclophosphamide	11	6/11 (55%)	0	1/11 (9%)	5/11 (45%)	4.8 m (range 1.5–8.3 m)	0
Ahmed et al., 2017 [26]	1	Glioblastoma	Progressive	HER2-specific CAR-T cell	no lymphodepletion	16	8/16 (50%)	0	1/16 (6%)	7/16 (44%)	NA	0
Zhang et al., 2017 [27]	1	Colorectal cancer	4	CEA-specific CAR-T cell	cyclophosphamide and fludarabine	10	7/10 (70%)	0	0	7/10 (70%)	NA	0
Katz et al., 2015 [28]	1	CEA positive liver metastasis	4	HAI of CEA-specific CAR-T cell	No lymphodepletion	6	1/6 (17%)	0	0	1/6 (17%)	NA	0
Ahmed et al., 2013 [29]	1/2	Sarcoma	R/R	HER2-specific CAR-T cell	No lymphodepletion	17	4/17 (24%)	0	0	4/17 (24%)	NA	0
Louis et al., 2017 [30]	1	Neuroblastoma	2-4	GD-2-specific CAR-T cell	No Lymphodepletion	11	5/11 (45%)	3/11 (27%)	1/11 (9%)	1/11 (9%)	NA	0

Guo et al. (2017) conducted a phase I clinical trial to evaluate the efficacy of EGFR-specific CAR-T cells in patients with relapsed/refractory stage 4 biliary tract cancers. Nab-paclitaxel in combination with cyclophosphamide was used for lymphodepletion. Out of 17 evaluable patients, 11 (65%) patients had objective responses, with one patient (6%) having a complete response for 22 months, and 10 (59%) patients had stable disease. The median progression-free survival was four months (range, 2.5-22 months). Grade 3 cytokine release syndrome was reported in only one patient who developed acute respiratory distress with pulmonary edema and improved after the administration of tocilizumab. Neurotoxicity ≥ grade 3 was not seen [16].

The phase I trial by Wallen et al. (2009) evaluated the efficacy of CAR cytotoxic T cells (CAR-CTL) specific to Mart-1, Gp 100, or tyrosinase antigen in the treatment of patients with stage 4 metastatic melanoma. Fludarabine was used for conditioning chemotherapy. At four-week intervals, three (33%) out of nine evaluable patients had objective responses, including one patient with a minor response (20%-30% reduction) and two patients (22%) with stable disease lasting for five-point eight (5.8) and seven months. However, out of six patients (67%) with progression at four-week intervals, two patients had delayed stabilization of disease and prolonged survival for 10.1 and 13.7 months. The median progression-free survival and the median overall survival were 2.1 months and 9.7 months, respectively. Grade ≥ 3 neurotoxicity or cytokine release syndrome was not reported [17].

Adusumilli et al. (2019) in a phase 1 trial used mesothelin-specific CAR-T cells in combination with anti-PD 1 checkpoint inhibitors for the treatment of primary (malignant mesothelioma) and secondary (metastatic breast and lung carcinoma) malignant pleural neoplasms. Second-generation CAR-T with the CD28 costimulatory domain and inducible caspase 9 safety gene were used. Cyclophosphamide was used for lymphodepletion. Out of 14 evaluable patients, 11 (79%) patients had objective responses. Two patients (14%) had complete responses lasting 62 and 39 weeks at the time of reporting of the study, five patients (36%) had partial responses, and four patients (29%) had stable disease. The six-month survival for this study was 100% and one-year survival was 80%. No events of ≥ grade 3 neurotoxicity or cytokine release syndrome were recorded [18].

Zhan et al. (2019), in a phase 1 clinical trial, used CAR-T cell therapy for the treatment of Claudin 18.2 positive advanced gastric and pancreatic adenocarcinoma. Claudin 18.2-specific CAR-T cells were used after patients underwent lymphodepletion with fludarabine and cyclophosphamide, with or without nab-paclitaxel. Out of 11 evaluable patients, nine (82%) patients had an objective response with complete response in one patient (9%), partial response in three (27%) patients, and stable disease in two (18%) patients. The median progression-free survival and the median overall survival were 136 days (range, 44-237 days) and 242 days (range, 55-349 days), respectively. No events of ≥ grade 3 neurotoxicity or cytokine release syndrome were recorded [19].

Becerra et al. (2019), in a phase 1 clinical trial, used the GoCAR-T cell product BPX-601 in patients with prostate surface cell antigen-positive metastatic pancreatic, gastric, and prostate cancers with measurable disease. The GoCAR-T technology combines antigen-specific CAR-T cells with inducible MyD88/CD40(IMC) in order to allow the control of T cell survival even in the absence of antigen. BPX-601 is an autologous T cell product expressing PSCA-CD3ζ CAR and a rimiducid (Rim)-inducible MyD88/CD40 co-activation switch to augment T-cell proliferation and persistence. Cyclophosphamide with or without fludarabine was used for lymphodepletion. Out of 13 evaluable patients, eight patients (62%) had objective responses with stable disease. The rest of the five patients (38%) had progressive disease. Median progression-free survival or overall survival was not reported. Grade 3 or 4 neurotoxicity of cytokine release syndrome was not reported [20].

Hegde et al. (2017) conducted a phase 1 clinical trial in patients with HER2 positive refractory and metastatic sarcomas using HER2-specific CAR-T cells. In these patients, autologous HER2-CAR-T cells with a CD28.zeta (CD28.ζ) signaling domain were used in combination with either fludarabine alone or fludarabine and cyclophosphamide for lymphodepletion. Out of six evaluable patients, four (67%) patients had objective responses, including two patients (33%) with a complete response and two patients with stable disease (33%). The median overall survival was 14.2 months. No events of ≥ grade 3 neurotoxicity or cytokine release syndrome were recorded [21].

The phase 1 clinical trial by Tanyi et al. (2016) used anti-mesothelin CAR-T cell therapy in patients with advanced and progressive stage 3 and 4 epithelial ovarian cancer. Autologous T cells transduced with lentiviral vectors to express anti-mesothelin chimeric antigen receptors fused with the intracellular signaling domain 4-1BB and TCR zeta (anti-mesothelin scFv TCRz:41BB). Lymphodepletion was achieved with cyclophosphamide. All six of the evaluable patients had stable disease at the time of evaluation, resulting in a 100% objective response rate. Median progression-free survival and medical overall survival were not reported. Greater than or equal to grade 3 neurotoxicity or cytokine release syndrome (CRS) was not reported [22].

Zhai et al. (2017), in a phase 1 clinical trial, evaluated the efficacy of anti-GP3 CAR-T cells in Chinese patients with refractory/relapsed glypican 3-positive hepatocellular carcinoma. Fludarabine, in combination with cyclophosphamide, was used for lymphodepletion. Out of six evaluable patients, four (67%) patients had objective responses, including one patient (17%) with partial response and three patients (50%) with stable disease. One patient with partial response remained alive for 385 days, two patients with stable disease remained alive at 384 and 562 days, respectively. One patient with stable disease died after 108 days. CRS or neurotoxicity equal to or greater than grade 3 was not reported [23].

Beatty et al. (2018) conducted a phase 1 trial to evaluate the efficacy of CAR-T cells in the treatment of metastatic chemotherapy-resistant pancreatic ductal adenocarcinoma. Mesothelin-specific CAR-T cells, including both the CD3-zeta and 4-1BB costimulatory domains, were used without lymphodepletion. Out of six evaluable patients, two (33%) patients had objective responses, with stable disease lasting for 3.8 and 5.4 months. CRS or neurotoxicity equal to or greater than grade 3 was not reported [24].

Feng et al. (2017) carried out a phase 1 clinical trial to evaluate the role of CAR-T cell therapy in the treatment of HER2-positive advanced biliary tract and pancreatic cancers. The study involved patients with intrahepatic and perihilar cholangiocarcinoma, pancreatic carcinoma, and gallbladder cancer. HER2-specific CAR-T cells with a CD8a hinge and the CD137 and CD3ζ signaling domains were given to the patients in addition to conditioning chemotherapy with nab-paclitaxel and cyclophosphamide. Out of 11 evaluable patients, six (55%) patients had objective responses, including one (9%) partial response for 4.5 months and five (45%) stable diseases. The median progression-free survival was 4.8 months (range, 1.5-8.3 months). CRS or neurotoxicity equal to or greater than grade 3 was not reported [25].

Ahmed et al. (2017) conducted a phase I clinical trial by using CAR-T cells for the treatment of HER2-positive progressive glioblastoma multiforme. HER2 CAR-T cells with the CD28 zeta signaling end domain were used without lymphodepletion. Out of 16 evaluable patients, eight (50%) patients had objective responses, including one patient (6%) with partial response for more than nine months and seven patients (44%) with stable disease for eight weeks to 29 months. Three patients with stable disease were alive at 29, 28.8, and 24 months of follow-up. The median overall survival was 11.1 months (95% CI, 4.1-27.2 months) from the first T-cell infusion and 24.5 months (95% CI, 17.2-34.6 months) from diagnosis. CRS or neurotoxicity equal to or greater than grade 3 was not reported [26].

Zhang et al. (2017) used carcinoembryonic antigen (CEA)-specific CAR-T cell therapy in the treatment of metastasized colorectal carcinoma. CAR-T cell therapy was used in combination with conditioning chemotherapy with cyclophosphamide and fludarabine. Out of 10 evaluable patients, seven (70%) patients had objective responses with stable disease. Two patients remained in stable disease for more than 30 months at the time of the conclusion of the study. CRS or neurotoxicity equal to or greater than grade 3 was not reported [27].

Katz et al. (2015) in their phase I clinical trial carried out hepatic arterial infusion of CAR-T cells for the treatment of carcinoembryonic antigen-positive liver metastasis. Intra-arterial infusion of anti-CEA CART- cells were done with and without interleukin-2 (IL-2). Conditioning chemotherapy was not used. Out of six evaluable patients, one (17%) patient had an objective response with stable disease. The patient with stable disease remained alive at 23 months at the time of the conclusion of the study. No patient reported grade 3 or 4 neurotoxicity of cytokine release syndrome [28].

Ahmed et al. (2013) conducted a phase 1/2 clinical trial to evaluate the efficacy of CAR-T cell therapy in the treatment of relapsed/refractory human epidermal growth factor 2-positive sarcomas. HER2-specific chimeric antigen receptor-specific T cells with the CD28.ζ signaling domain were used with conditioning chemotherapy. Out of 17 evaluable patients, four (24%) patients had objective responses with stable disease for 12 weeks to 14 months. The median overall survival was 10.3 months (range, 5.1 to 29.1 months). No patient reported grade 3 or 4 CRS or neurotoxicity [29].

Louis et al. (2011) used CAR-T cell therapy in this phase 1 clinical trial to evaluate its efficacy in the treatment of progressive GD-2 positive neuroblastoma. Chimeric antigen receptors specific to GD-2 antigen were expressed on cytotoxic T cells (CAR-CTL) and activated T cells (CAR-ATC). CAR-CTL and CAR-ATC specific to GD-2 antigen were used without conditioning chemotherapy. Out of 11 evaluable patients with relapsed/refractory progressive disease, five (45%) patients had objective responses at six weeks, including two patients with complete response (18%), one patient with partial response (9%), and two patients (18%) with stable disease. No patient reported grade 3 or 4 CRS or neurotoxicity [30].

Discussion

Our literature review constituted early phase 1 clinical trials that are not designed to evaluate efficacy, but it does show some promise in terms of good response rates in some of the cancers.

The majority of the patients achieved an overall response and/or clinical benefit in a phase 1 trial evaluating ovarian epithelial cancer (100%), HER2-positive sarcoma (67%), EGFR-positive biliary tract cancer (65%), advanced gastric/pancreatic cancer (82%), hepatocellular carcinoma (67%), and colorectal cancer (70%). Although the cross-trial comparison is less than ideal, historically, the response rates in the second-line setting in each respective cancer have been much lower. For example, in platinum-refractory relapsed ovarian cancer patients, the response rate was around 20% with single-agent chemotherapy and 31% in combination with VEGF receptor antibody bevacizumab [31-33]. Similarly, in relapsed metastatic pancreatic cancer, a nanoliposomal irinotecan and 5FU-based regimen in gemcitabine refractory patients had a response rate of 16% [34]. Glioblastoma is a highly aggressive cancer with a median overall survival of six months in the relapsed setting [35]. In the phase 1 trial by Ahmed et al., we saw prolonged survival in glioblastoma multiforme (GBM) patients utilizing autologous HER2 CMV bispecific CAR-T cells for progressive GBM. Out of five patients that responded, three patients were noted to have prolonged survival lasting more than 24 months and were still alive at the time of reporting results [26].

Cytokine release syndrome and neurotoxicity were the most serious adverse events associated with CD-19 specific CAR-T therapy and were graded according to the National Cancer Institute Common Terminology Criteria for Adverse Events (CTCAE) [36-37]. However, in our studies with solid tumors, no event of grade 3 or 4 neurotoxicity was reported with CAR-T administration and one episode of grade 3 cytokine release syndrome was reported with acute respiratory distress that required tocilizumab injection with EGFR-specific CAR-T cells in biliary tract cancers [16]. The use of high-intensity conditioning chemotherapy in hematological malignancies has shown to increase T cell persistence and efficacy and has shown beneficial response in decreasing the incidence and severity of cytokine release syndrome [38]. Cyclophosphamide, fludarabine, and nab-paclitaxel either alone or in combination were used in most of the studies. Conditioning chemotherapy was not used in association with CAR-T cell therapy in clinical trials involving glioblastoma multiforme and neuroblastomas [26,30]. Despite approval in some hematological malignancies, CAR-T cell therapy has a long way to go in solid tumor management. Solid tumors present different sets of challenges that need to be addressed in order for CAR-T cell therapy to be effective. For instance, in hematological malignancies, such as B-cell lymphomas, CD19 antigen on B-cells is the most commonly employed target by CAR-T cell therapy and the most common side effects are B-cell aplasia and hypogammaglobulinemia [39], whereas solid tumors are more heterogeneous and present difficulty in antigen selection. The ideal antigen for CAR-T cell therapy should be confined to tumor cells and be absent from normal cells. In reality, the majority of antigens that are present in high concentrations in tumor cells are also present in normal tissue cells that lead to on-target, off-tumor toxicity [40]. CAR-T cells that target multiple antigens within a tumor help overcome the on-target, off-tumor toxicity [41]. Another major challenge is the trafficking of the cytotoxic T cells to the solid tumor that is a highly regulated process and is typically altered by a mismatch of chemokine-chemokine receptors, downregulation of adhesion molecules, and abnormal vasculature by the tumor cells or stroma [42]. CAR- T cells expressing tumor-specific chemokine receptors improve the trafficking to the site of the tumor [43-45]. Similarly, in the clinical trial by Katz et al., localized administration of CAR-T cells through hepatic arterial infusion was employed to overcome the challenges of trafficking of T cells to the site of the tumor [28].

The tumor microenvironment has physical barriers and immunosuppressive cytokines that inhibit not only the infiltration of T cells but also their ability to exert an antitumor effect. CAR-T cells were modified to express enzymes that can degrade extracellular matrices in the tumor stroma in mice models to improve the infiltration and prolongation of T cell survival [46]. The tumor cells or surrounding stromal cells secrete cytokines such as programmed cell death protein 1 (PD1) and cytotoxic T-lymphocyte-associated antigen 4 (CTLA4) to inhibit T cell functions [47]. Inhibition of these cytokines enhances the antitumor effect of tumor-infiltrating lymphocytes and improves clinical outcomes [48-49]. For instance, Adusumilli et al. used CAR-T cell therapy in combination with anti-PD1 checkpoint inhibitors to improve the efficacy of T cells in the treatment of malignant pleural tumors [18].

## Conclusions

In conclusion, CAR-T cell therapy in solid malignancies is still in the initial phase of development. Phase 1 data across various solid cancers shows that it is not only safe but also has encouraging results in terms of overall response rates. It is unclear how sustained these responses are, and survival data are immature. It would be interesting to see if combining immunotherapy could augment and prolong response with CAR-T cell therapy. Patients should be encouraged to participate in CAR-T clinical trials whenever available.
